# Availability of food resources and habitat structure shape the individual‐resource network of a Neotropical marsupial

**DOI:** 10.1002/ece3.5024

**Published:** 2019-03-21

**Authors:** Nícholas F. de Camargo, Hernani F. M. de Oliveira, Juliana F. Ribeiro, Amabílio J. A. de Camargo, Emerson M. Vieira

**Affiliations:** ^1^ Laboratório de Ecologia de Vertebrados, Departamento de Ecologia, Instituto de Ciências Biológicas Universidade de Brasília Brasília Brazil; ^2^ Department of Ecology Faculty of Science, Charles University Viničná Prague Czechia; ^3^ Embrapa Cerrados Planaltina Brazil

**Keywords:** Cerrado, modularity, nestedness, population, seasonality

## Abstract

1. Spatial and temporal variation in networks has been reported in different studies. However, the many effects of habitat structure and food resource availability variation on network structures have remained poorly investigated, especially in individual‐based networks. This approach can shed light on individual specialization of resource use and how habitat variations shape trophic interactions.
2. To test hypotheses related to habitat variability on trophic interactions, we investigated seasonal and spatial variation in network structure of four populations of the marsupial *Gracilinanus agilis* in the highly seasonal tropical savannas of the Brazilian Cerrado.
3. We evaluated such variation with network nestedness and modularity considering both cool‐dry and warm‐wet seasons, and related such variations with food resource availability and habitat structure (considered in the present study as environmental variation) in four sites of savanna woodland forest.
4. Network analyses showed that modularity (but not nestedness) was consistently lower during the cool‐dry season in all *G. agilis* populations. Our results indicated that nestedness is related to habitat structure, showing that this metric increases in sites with thick and spaced trees. On the other hand, modularity was positively related to diversity of arthropods and abundance of fruits.
5. We propose that the relationship between nestedness and habitat structure is an outcome of individual variation in the vertical space and food resource use by *G. agilis* in sites with thick and spaced trees. Moreover, individual specialization in resource‐rich and population‐dense periods possibly increased the network modularity of *G. agilis*. Therefore, our study reveals that environment variability considering spatial and temporal components is important for shaping network structure of populations.

## 1 INTRODUCTION

The relevance of interactions among organisms for community stability represents one of the pivotal issues in ecology (May, 1972; McCann, 2000; Neutel, Heesterbeek, & de Ruiter, 2002). To summarize interactions among species that are often complex and dynamic, network approaches have been widely used as a powerful method. For that, species may be represented by nodes and interactions by links between nodes (van Veen, Müller, Pell, & Godfray, 2008), and to quantitatively describe the network structure, different metrics have been proposed (e.g., Vázquez, Chacoff, & Cagnolo, 2009). This analytical approach allowed, for example, to compare network patterns between different types of interactions (e.g., mutualistic or antagonistic links) (Lewinsohn, Prado, Jordano, Bascompte, & Olesen, 2006; Nuwagaba, 2015; Thébault & Fontaine, 2010), to comprehend how habitat and climate change and species extinction affect network structure (Gilman, Urban, Tewksbury, Gilchrist, & Holt, 2010; Tylianakis, Laliberté, Nielsen, & Bascompte, 2010; Valiente‐Banuet et al., 2015), and to understand coevolutionary dynamics (Jordano, Bascompte, & Olesen, 2003; Rezende, Lavabre, Guimarães, Jordano, & Bascompte, 2007; Wade, 2007).

Most networks of interactions are usually built at the community‐level enclosing many species (Pocock, Evans, & Memmott, 2012; Wirta, Weingartner, Hambäck, & Roslin, 2015). However, although studies recognize that individual variation is a relevant driver for intra‐ and interspecific competition and for the structure and dynamics of ecological networks (Bolnick et al., 2010; Bolnick, Yang, Fordyce, Davis, & Svanbäck, 2002; Cantor, Pires, Longo, Guimarães, & Setz, 2013; Svanbäck & Bolnick, 2005), there are still few studies focusing on within‐population patterns applying network approaches (but see Araújo et al., 2008; Araújo et al., 2010; Pires et al., 2011; Cantor et al., 2013; Lemos‐Costa, Pires, Araújo, Aguiar, & Guimarães, 2016). In fact, studies have showed that generalist populations may be comprised by relatively specialized individuals (Araújo et al., 2008; Bolnick, Svanbäck, et al., 2002; Svanbäck & Bolnick, 2007). Therefore, exclusive evaluation of species‐level networks can hide, for example, highly specialized interactions in generalists species that can be better evaluated on individual‐level networks (Tur, Vigalondo, Trøjelsgaard, Olesen, & Traveset, 2014).

Among the many proposed metrics that describe network structure, modularity, and nestedness have remained the most relevant ones to reveal changes in species interaction patterns and resource use (Fortuna et al., 2010; Olesen, Bascompte, Dupont, & Jordano, 2007; Thébault & Fontaine, 2010). Previous studies have showed that both metrics are important to represent interactions among individual consumers of a population and different types of food resources (Araújo et al., 2008, 2010; Pires et al., 2011). These studies state that on the within‐population networks context, if individuals of a population present different diet preferences, they might be organized in distinct groups formed by individuals specialized on distinct sets of resources, generating a modular network (Araújo et al., 2008; Pires et al., 2011). On the other hand, nestedness emerges if the individuals have a differentiated degree of selectivity, in which selective individuals feed on subsets of the broader diet of the generalist individuals (Araújo et al., 2010; Pires et al., 2011). Studies investigating the structure for populations of different vertebrate taxa have showed that nested networks are more common than modular networks, suggesting that these populations are formed by both opportunistic and selective individuals (Pires et al., 2011). Moreover, it has been suggested that variations on prey preferences between individuals are the main factors explaining changes in individual‐resource networks (Lemos‐Costa et al., 2016; Pires et al., 2011).

Three different models were proposed to explain individual diet specialization within populations (Svanbäck & Bolnick, 2005). The “shared preference model” states that, as individuals present identical rank of preferable food items, and these populations are composed by specialists and generalists, new resources are added in a predictable order producing nestedness. On the other hand, the “distinct preference model” assumes that individuals differ in the rank of resource preference order. However, whereas strong individual specialization occurs at low population density, it declines at high population density since competition leads to an expansion of the individuals' diet. Lastly, the “competitive refuge model” assumes that individuals share top‐ranked resources but differ in the choice of the alternative ones. According to this model, a lack of individual specialization occurs during low forager densities and increases as preferred resources become less available due to increasing forager densities.

Despite the advances on identifying the patterns of within‐population networks, the influence of abiotic and biotic factors in shaping network structure has remained largely unexplored, and still represents a frontier for the comprehension of network dynamics (Bascompte & Jordano, 2007). Environments with high within‐year variation in resource availability and habitat structure are adequate for testing hypotheses related to the effects of seasonal changes in biotic and abiotic factors on trophic interactions within populations. This is the case of the highly seasonal Neotropical savanna—the Cerrado, which presents well‐defined cool‐dry and warm‐wet seasons (Eiten, 1972). Therefore, food resources availability can vary between seasons (Gouveia & Felfili, 1998; Pinheiro, Diniz, Coelho, & Bandeira, 2002; Silva, Frizzas, & Oliveira, 2011), as well as the microhabitat structure (e.g., herbaceous and canopy cover) due to the expansion and the retraction of the vegetation biomass (Schwieder et al., 2016). In this Neotropical savanna, several mammal species present between‐season differences in both diet and space use (Camargo, Ribeiro, Camargo, & Vieira, 2014a, 2014b; Hannibal & Caceres, 2010; Lessa & da Costa, 2010; Ribeiro, 2011; Vieira, 2003). Therefore, the Cerrado systems provide valuable opportunities for the evaluation of how seasonal variation in resources availability and habitat structure affects patterns of within‐population network structure. Considering that even localities with the same vegetation type in Cerrado can present local‐scale differences between sites in relation to habitat structure and food availability (Camargo et al., 2014a; Mendonça et al., 2015), investigating distinct populations of the same species can help to elucidate how these biotic factors locally shape the network interactions according to nestedness and modularity.

In the present study, we investigated seasonal and spatial variation in individual‐based network structure of the didelphid marsupial *Gracilinanus agilis* (Burmeister, 1854) in four distinct populations within the Brazilian Cerrado. We evaluated if changes in nestedness and modularity between populations are explained by spatial and temporal differences in food resources availability and habitat structure (hereafter referred as environmental variation) in sites of savanna woodland forest (locally known as cerradão). We expected higher values of nestedness to occur during the warm‐wet season due to the high resource availability in this season (Araújo et al., 2010; Cantor et al., 2013). This is expected because in periods with higher abundance and richness of fruits and arthropods (Gouveia & Felfili, 1998; Pinheiro et al., 2002; Silva et al., 2011), there is a decrease in dietary overlap between those individuals with broad (generalists) and those with narrow diet (specialists), increasing the degree of network nestedness (as in Cantor et al., 2013). Therefore, considering populations of distinct sites and seasons, we expected a positive relationship between nestedness and food resource availability.

Since modularity may increase with habitat complexity (Macfadyen, Gibson, Symondson, & Memmott, 2011; Pimm & Lawton, 1980; Rezende, Albert, Fortuna, & Bascompte, 2009), we also expected an increase of modularity in the warm‐wet season. More specifically, we expected that with the biomass increasing of the vegetation during the rainy season (Schwieder et al., 2016), new microhabitats would be available for groups of individuals to exploit their resources, generating modules. Therefore, we also expected a positive relationship between habitat structure related to vegetation density and modularity considering the four distinct populations studied in both cool‐dry and warm‐wet seasons.

## 2 MATERIALS AND METHODS

### 2.1 Studied species

The gracile mouse opossum *G. agilis* is a small (20–30 g of body mass), solitary, nocturnal and scansorial marsupial whose distribution ranges from the border of Panama with Colombia to the Northeast, Midwest, and Southeast of Brazil (Emmons & Feer, 1997). Generally common in forest formations present in the Brazilian Cerrado (Nitikman & Mares, 1987), this marsupial has a seasonal pattern of reproduction, with females in reproductive condition from the last month of the cool‐dry season to the middle/end of the warm‐wet season (Martins, Bonato, Da‐Silva, & Dos Reis, 2006). The diet of *G. agilis* is comprised mainly by pioneer fruits and several orders of arthropods, but occasionally this species feeds on birds (Camargo et al., 2014a).

### 2.2 Study area

We conducted our study in the core area of the Cerrado, the second largest biome of South America (Ab'Sáber, 1977). The Cerrado is characterized by two well‐marked cool‐dry and warm‐wet seasons, with the later occurring between October and April, when 90% of the annual precipitation of 1,100–1,600 mm occurs (Miranda, Miranda, & Dias, 1993). Vegetation types include typical savanna habitats, grasslands, and forests, that are influenced by edaphic features (Ribeiro & Walter, 1998). One of the forest types occurring in the Cerrado is the savanna woodland (locally known as cerradão), a xeromorphic forest formation with trees that range from 8 to 15 meters and a tree layer that oscillates between 50% and 90% (Ribeiro & Walter, 1998).

**Figure 1:**
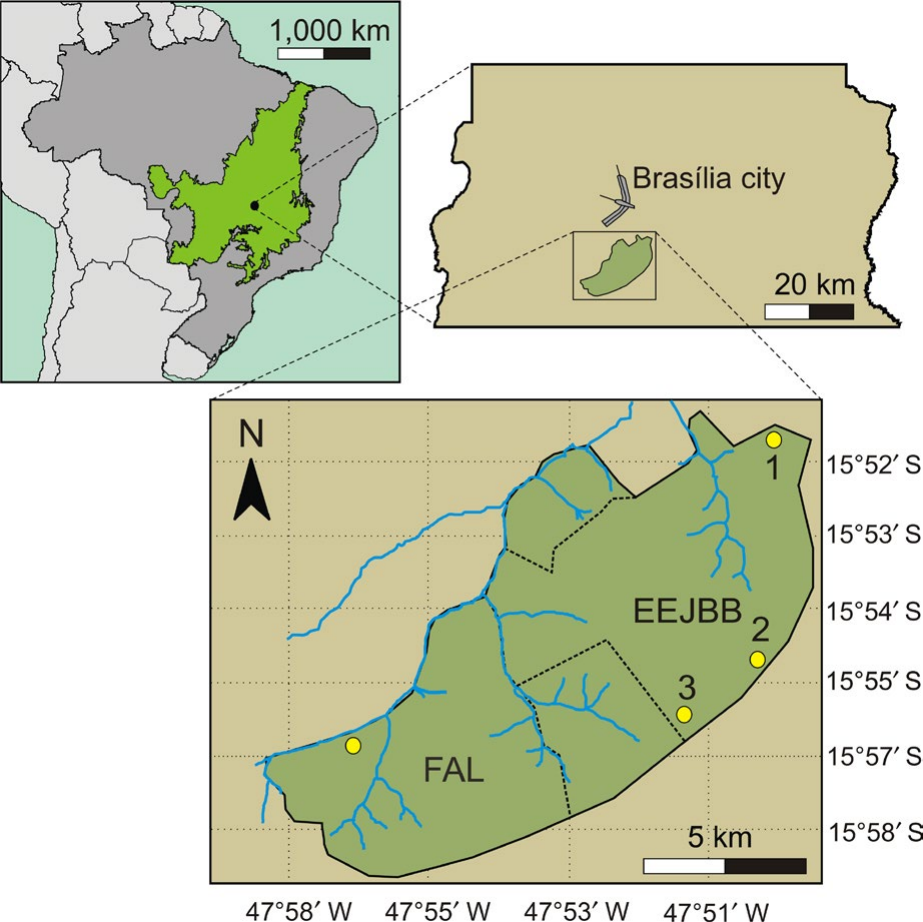
Location of the study sites in the neotropical savanna (Cerrado), showed as a green area in the Brazil's map (dark gray area) on the top left. These sites were located near the city of Brasília in the Federal District of Brazil (top right inset). The bottom map indicates the detailed location of the four sampled sites of cerradão (savanna woodland forest), three at the Botanical Garden of Brasília (EEJBB1, EEJBB2, and EEJBB3) and one fragment at the ecological and agricultural field station of the University of Brasília (FAL)

Our data collection was conducted between 2009 and 2010 in four sites of savanna woodland forest near the city of Brasília, the Federal District of Brazil. These sites were located at the Ecological Station of the Botanic Garden of Brasília (EEJBB in Portuguese; 15°52′S, 47°50′W) and Fazenda Água Limpa, the ecological and agricultural field station of the University of Brasília (FAL in Portuguese; 15°58′S, 47°59′W (Figure 1). These two locations are part of the Area of Environmental Protection (APA) Gama e Cabeça‐de‐Veado, which covers about 15,000 ha of continuous Cerrado vegetation.

### 2.3 Capture procedures and identification of food items

We captured *G. agilis* using Sherman live traps placed in four grids, each one located in a distinct dry‐woodland site. Three grids were located at EEJBB (EEJBB1, EEJBB2, and EEJBB3) and one grid located at FAL (Figure 1), each one composed of 144 (12 × 12) capture stations spaced at 15‐m intervals. In each capture session and for each grid, we randomly selected 80 capture stations for placing traps on the ground and 80 capture stations for placing traps on the understory (1.5–2.5 m high). All the grids were sampled three times in each season during six consecutive nights, totaling 23,040 trapping‐nights. As bait, we used a uniform mixture of peanut butter, corn flour, mashed banana, cod liver oil, and vanilla essence. Each captured individual received a numbered ear‐tag (National Band and Tag Co., Newport, Kentucky, USA, Monel tag, size 1) for further identifications.

We collected scats for diet evaluation from traps or during handling of the trapped animals. These scats were analyzed in laboratory, and food categories were identified at the lowest possible taxonomic category by comparison with a reference collection of invertebrates and fruits from the study area. Details on fecal analysis can be found in other studies based on the same database that we used in the present study (Camargo et al., 2014a; Camargo, Ribeiro, Camargo, & Vieira, 2014b). Fecal analysis is considered an effective method for assessing the diet of Neotropical marsupials (Araújo et al., 2010; e.g., Pires, Martins, Araujo, & Reis, 2013; Camargo et al., 2014b), despite some intrinsic limitations of the method, such as differential digestibility of food items, potential problems for the identification of food items at lower taxonomic level (e.g., family or genus), and difficulty in estimating the relative importance of each food item (Araújo et al., 2010). Differently of other studies that estimated the number of items consumed based on small fecal remains (the number of insects consumed based on the number of leg pieces, or the number of fruits based on seed and fiber count; Anthony & Kunz, 1977; Mallet‐Rodrigues, 2001; Pires et al., 2013), we opted for a conservative approach of considering only the occurrence of each food category.

### 2.4 Food resource availability

For assessing food availability for *G. agilis* during the warm‐wet and cool‐dry seasons in each site and each capture session, we estimated the availability of arthropods and fruits. For the evaluation of arthropod availability, we set 30 pitfalls at each grid in each capture session for three consecutive days. These traps consisted of 200‐ml plastic cups buried with the opening flush to the ground surface. Each trap was filled with formaldehyde, water, and drops of soap to break the water tension. In each capture session, these pitfalls were arranged in three transects that were randomly distributed within the grids, always avoiding repeating the local in which transects were placed. We identified the collected arthropods up to order level and obtained the availability of this resource measuring the biomass to the nearest 0.0001 g (dry weight) of each arthropod order per sampling session. For that we dried all the collected arthropods in an oven at 60°C for 72 hr. Similarly, for obtaining the availability of fruits during the cool‐dry and warm‐wet seasons in each site, in each capture session we randomly established eight transects with 20 × 5 m in which we counted the fruits on plants.

### 2.5 Measurements of habitat structure

Concomitantly with the period of *G. agilis* captures, we obtained descriptions of habitat structure in the warm‐wet and cool‐dry seasons by measuring eight structural variables in randomly selected capture stations. The number of stations sampled ranged from 57 to 78 per season in each site, distributed as following (site: number of samples in the cool‐dry, number of samples in the warm‐wet season): FAL: 61, 76; EEJBB1: 60, 65; EEJBB2: 57, 68; EEJBB3: 71, 78). For the evaluation of habitat structure, each capture station was divided into four quadrants and the measurements were taken: (a) understory obstruction at 1.5 m height, which was estimated using a polyvinyl chloride (PVC) square of 0.25 m^2^ (0.50 × 0.50 m) divided into 50 open squares with a nylon mesh (see Freitas, Cerqueira, & Vieira, 2002 for more details); (b) herbaceous obstruction also measured with the same PVC square; (c) percentage of canopy openness measured with a concave densitometer positioned at 1.5 m height (Lemmon, 1956); (d) canopy connectivity (Freitas et al., 2002); (e) litter depth using a measuring tape; (f) diameter at breast height (dbh) of the nearest tree with minimum diameter of 16 cm; (g) distance to the nearest tree with dbh >16 cm; and (h) height of the nearest tree with dbh >16 cm. These microhabitat variables are potentially relevant for the occurrence of small mammals (Camargo et al., 2018; Mendonça et al., 2015) and also describe heterogeneity and complexity variation of the habitat. For each variable, we calculated the average value considering all the measurements obtained in the four quadrants. For further analyses, to remove scale effects among variables, values for each variable were autoscaled using Z transformation (Zar, 1999).

### 2.6 Data analysis

#### 2.6.1 Network analyses

We used the Chao1 estimator of richness to assess whether through the fecal samples of *G. agilis* collected in field, we were able to detect most of the food items that could potentially be consumed by the marsupial (e.g., Dalsgaard et al., 2017). We then used the dietary information to generate individual‐resource networks in which consumer nodes refer to individuals and resource nodes represent food resource categories. To calculate nestedness, we used the method BINMATNEST from the function network level of the Bipartite package (Dormann, Gruber, & Fründ, 2008) in the software R (R Development Core Team, 2017). This algorithm reorders the rows and columns leading to a minimum matrix temperature and then calculates the statistical significance of matrix temperature (Rodríguez‐Gironés & Santamaría, 2006). A matrix temperature is a measure of how much the incidence matrix diverges from a perfected nestedness (Almeida‐Neto, Guimarães, & Lewinsohn, 2007). For the calculation of modularity, we used the community detection algorithm fast greedy (Newman & Girvan, 2004) using the package igraph (Csardi & Nepusz, 2006). This algorithm calculates modularity according to a maximization function, where the division of the network in modules is based on the higher density of connections inside modules than among them (Guimerà, Sales‐Pardo, & Amaral, 2007; Newman & Girvan, 2004). Thus, this method quantify whether within‐module interactions are more prevalent than between‐module interactions (Dormann & Strauss, 2014). Both network nestedness and modularity were obtained considering the food items found in the fecal samples of *G. agilis*during the cool‐dry and warm‐wet seasons separately in each site.

We also calculated the connectance of the studied networks, a metric commonly used to characterize specialization in species‐level networks (e.g., Olesen & Jordano, 2002; Devoto, Medan, & Montaldo, 2005). This metric is defined as the proportion of the observed interactions to all possible interactions (Fortuna et al., 2010), and under the population‐level network context, would indicate the degree of individual specialization in the network. However, the results indicated that this metric was highly correlated to modularity (Spearman correlation; *r* = −0.86, see Supporting Information Table S1). Thus, we decided to present only the results related to the latter. Complementarily, we also assessed the degree distribution (i.e., the number of connections of a node; Bollobás, 1998), which was obtained by calculating the proportion of individuals that interacted with *n* food items. The degree distribution in population‐level networks would indicate whether, in general, individuals tend to interact more or less with different food items in each site considering both seasons.

In order to test for the significance of nestedness and modularity, we used the Erdõs‐Rényi model (Erdõs & Rényi, 1959), which generates networks with the same size and connectance as the observed network, but with a random distribution of the links where the probability of two nodes (marsupial – prey) to have a connection is 50%. This model guarantees that all food items have the same probability to be selected or not be selected at random. We built 1,000 randomized matrices and tested the significance of the observed values using a Monte Carlo procedure to infer whether these values differed than expected by chance (*α* = 5%).

We also calculated the standardized effect size (SES) to make results directly comparable across sites as well as with other studies. SES is defined as follows:

$$\text{SES}=\left({\text{Metric}}_{\text{obs}}-{\text{Mean}\;\text{of}\;\text{Metric}}_{\text{sim}}\right)/{\text{standard}\;\text{deviation}\;\text{of}\;\text{Metric}}_{\text{sim}},$$

where Metric_obs_ is the observed value for the given metric (nestedness or modularity) and Metric_sim_ are the simulated values for the metric. With a normal distribution of SES, the 95% confidence interval should range between 2 and −2 so that observed SES above 2 indicates that the correspondent metric is significantly higher than expected by chance and below −2 indicates that the correspondent metric is significantly lower than expected by chance.

#### 2.6.2 Habitat structure and food resources

For the evaluation of food resources availability, we used the total dry biomass of arthropods and the estimated diversity through the Shannon diversity index (Exp [*H*′]; Jost, 2006) considering the total dry mass of each order. These two metrics were tested independently considering indexes of nestedness and modularity obtained in each season for each site as dependent variables in linear regressions. Thus, we were able to evaluate whether there is a relationship between network metrics and food resources availability. We investigated the effect of fruit availability on the same network metrics in the same way, using as independent variable the total amount of fruits counted in each season in each site, which was log‐transformed to improve data normality. Preliminary investigation indicated that there was not any significant correlation between resource availability variables (arthropod diversity, arthropod biomass, and fruit abundance; Spearman's rank correlation coefficient, *p* > 0.1 for all comparisons).

For evaluating the relationship between habitat structure and network metrics, we first performed a principal component analysis (PCA) to produce two new variables (PC 1 and PC 2) that summarized most of the variation (>50%) of the habitat variables during the cool‐dry and warm‐wet seasons in each site. After conducting this PCA, we used the average scores of the first two axes for running simple linear regression analyzes considering PC 1 and PC 2 as independent variables and nestedness and modularity as dependent variables. These regressions were run independently for each PCA axis and network metrics. Analyses regarding linear regression and PCA were conducted using the software PAST v. 3.01 (Hammer, Harper, & Ryan, 2001).

Our study was based on the well‐established marked seasonal differences in food resource availability and habitat structure in the Cerrado (e.g., Gouveia & Felfili, 1998; Pinheiro et al., 2002; Schwieder et al., 2016) in order to obtain data with enough variation for detection of any possible network change. In fact, in our study we also found evidences of between‐season environmental variation (see Supporting Information Figures S1–S3). This approach allowed us to evaluate how networks of interactions changes as a population experiences environmental variation, and whether these network changes are predictable. Our sampling units regarding different seasons within a site, however, cannot be considered as independents in a strict statistical sense.

In our study, we used only one sample of each individual per season (warm‐wet and cool‐dry) to improve statistical independence of the samples. The network analyses were conducted based on 374 fecal samples of 319 individuals, distributed as following (site: number of samples of the cool‐dry season, number of samples of the warm‐wet season): FAL: 44, 72; EEJBB1: 36, 52; EEJBB2: 48, 58; EEJBB3: 37, 27. The proportion of fecal samples from recaptures in both season ranged from 11% to 19%.

## 3 RESULTS

### 3.1 Network structure

We found 20 distinct food items in the scats of *G. agilis*, represented by 10 arthropod orders (nine of insects and one of arachnid), pulp, fiber, and seeds of three plant families (represented by four species), three morphotypes of unidentified fruit fibers, and bird remains (feathers and bones) (for more details see Camargo et al., 2014a, 2014b). According to Chao1 estimator of richness, we detected between 67% and 100% of the food items that could potentially be consumed by *G. agilis* (Supporting Information Figure S4). Our results regarding the degree distribution showed that, in all sites, a high proportion of individuals tend to interact with more food items in the cool‐dry season (3 to 4 food items; between 37% and 48% of the individuals) than in the warm‐wet season (2 food items; between 42% and 61% of the individuals) (Supporting Information Figure S5).

**Figure 2:**
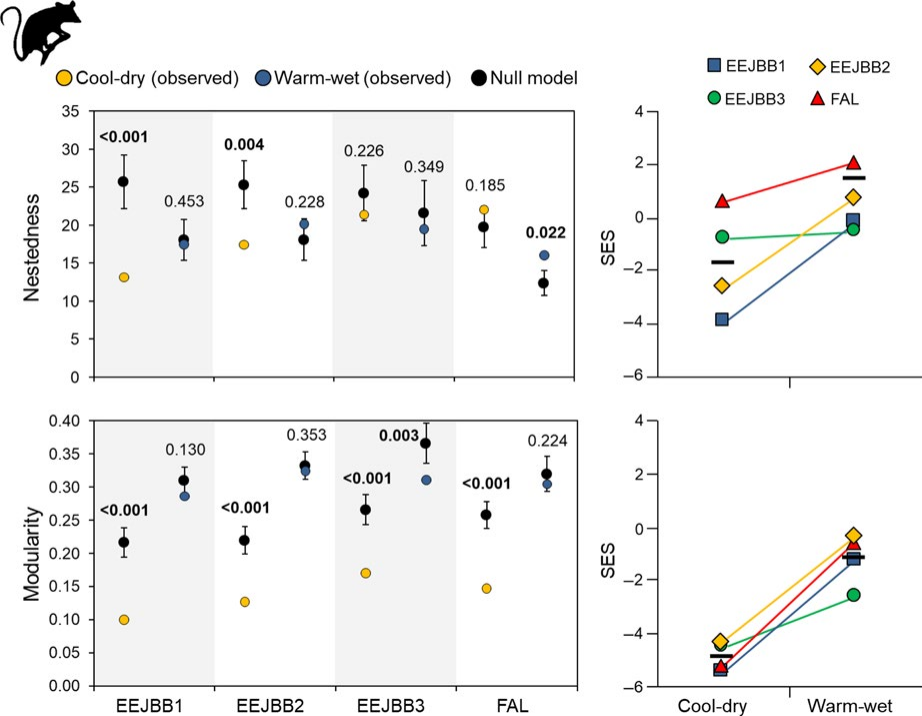
Observed and expected values of network nestedness and modularity of *Gracilinanus agilis* populations in four sites of savanna woodland forest (cerradão) in the Brazilian savanna (Cerrado). Black circles in the left graphics indicate the average expected values based on 1,000 runs for random networks. Vertical bars indicate the standard deviation of the simulated values. Bold values indicate probabilities (*p*) of the simulated distributions being different than expected by chance (*p* < 0.05). Graphics on the right indicate the Standard Effect Size (SES) from the null‐model analysis for the correspondent network metric of each site (horizontal mark indicates the mean value for each season considering the four sites)

Our results indicated that the four sites did not respond in the same way regarding to seasonal changes in nestedness. The null model indicated that both EEJBB1 and EEJBB2 presented networks less nested than expected by chance during the cool‐dry season, but during the warm‐wet season only in one site (FAL) the network was more nested than expected by chance. The mean SES was lower during the cool‐dry season, but this reduction was not similar for all sites (Figure 2).

In relation to modularity, the observed patterns for the four sites were more similar. During the cool‐dry season, Monte Carlo procedures showed that modularity was always lower than expected by chance. During the warm‐wet season, however, modularity did not differ from the expected by chance only in one site (EEJBB3), where observed values were lower than expected by chance (Figure 2). These between‐season differences were evident in the SES results, which showed that during the dry‐cool season the four sites had SES for modularity lower than during the warm‐wet season.

### 3.2 Environmental variation and network metrics

**Figure 3:**
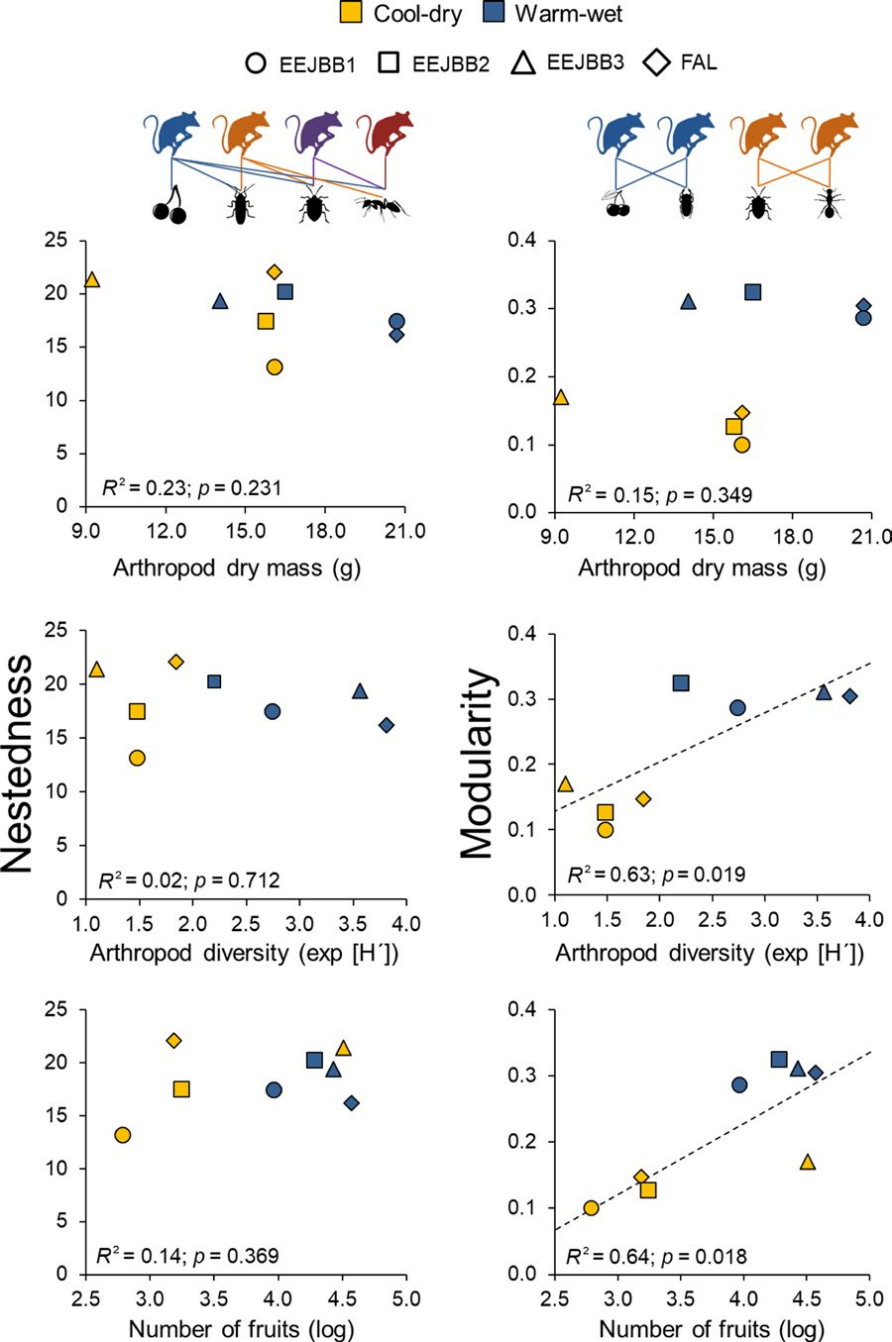
Relationship between nestedness and modularity with resource availability (linear regressions) obtained during the cool‐dry and warm‐wet seasons in four savanna woodland forest (cerradão) sites in the Brazilian savanna (Cerrado). Arthropod dry mass corresponds to the total amount of arthropods in each site and in each season obtained using pitfalls. Arthropod diversity (Shannon index – exp [*H*′]) was calculated using relative dry mass of each order. Number of fruits corresponds to the total counts of this food resource in transects in each site and in each season. Trend lines are shown only for the significant relationships (*p* < 0.05). Above the graphics are representations of nested (left) and modular networks (right)

In relation to resource availability and network metrics, both arthropod diversity (Shannon diversity index) and fruit abundance (obtained by fruit counting) were positively related to modularity (Figure 3). On the other hand, arthropod abundance (considering the total arthropod dry mass obtained per season) in each site showed no association with nestedness or modularity. Moreover, nestedness was not related to either arthropod diversity or fruit abundance.

Considering the habitat structure, the 1st component of the PCA explained 30.9% of the variance and the 2nd one 21.0%. The first axis was loaded most heavily (absolute factor loading ≥0.5) by canopy openness, canopy connectivity, tree diameter, and tree height, with only canopy openness of these four being negatively associated with this axis. Therefore, the PC 1 indicated, from negative to positive values, a gradient of sites that present more open to more closed vegetation (Supporting Information Table S2 and Figure S6). The second axis was more associated to tree diameter and distance to the nearest tree, with both variables negatively associated with PC 2. Therefore, this axis indicated, from negative to positive values, a gradient of sites that presented more spaced and larger diameter trees to sites with less spaced and smaller diameter trees (Supporting Information Table S2 and Figure S6).

**Figure 4:**
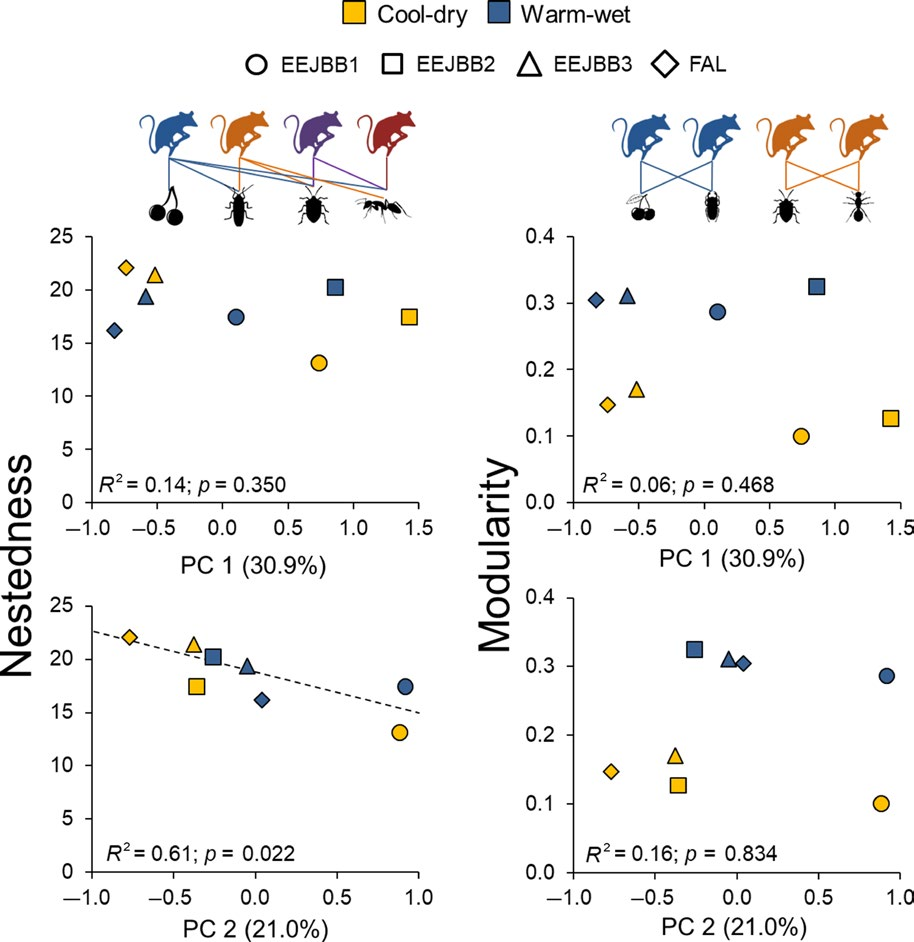
Relationship between nestedness and modularity with habitat structure (linear regressions) obtained during the cool‐dry and warm‐wet seasons for four sites of savanna woodland (cerradão) in the Brazilian savanna (Cerrado). PC 1 and PC 2 correspond to the first two axes obtained in a principal component analysis of eight structural habitat variables (see methods for more details). Trend lines are shown only for the significant relationships (*p* < 0.05). Above the graphics are representations of nested (left) and modular networks (right)

Our analysis regarding the relationship between network nestedness and modularity with the PC 1 showed no significant associations. For the second axis (PC 2), however, we found a significant and negative relationship with nestedness, indicating that nestedness increases in sites with more spaced and tick trees. Modularity showed no association with the PC 2 (Figure 4).

## 4 DISCUSSION

Our study on four distinct populations of *G. agilis* showed that their networks change seasonally and spatially. Network metrics were related to the variation of environmental factors, since the higher availability of arthropods (diversity) and fruits (abundance) during the warm‐wet season increased modularity, and nestedness increased in sites with mores spaced and thick trees. To our knowledge, this is the first study to assess how food resource availability and features of habitat structure directly affect interaction networks, evaluating simultaneously multiple populations also considering variation between seasons.

Patterns of network nestedness differed across sites for *G. agilis* populations, indicating that changes in nestedness between seasons were not similar considering each site. Contrarily to our initial expectation of a relationship between nestedness and food resource availability, our results revealed that this network metric is associated with habitat structure. Therefore, these results suggest that spatial variation of environmental components is important for local network structure. However, it is not clear to us why nestedness, for example, was lower than expected by chance in EEJBB1 and EJBB2 in the cool‐dry season, since we found no differences in habitat structure considering seasons in the PC 2 axis. It seems that other factors besides habitat structure were affecting nestedness in these sites.

Nestedness, on the light of individual‐resource network, can be considered an outcome of individual variation in the food resource use, which is resulted from a diet overlap between individuals with narrow dietary niches with those of broad dietary niches in the population. Previous studies with neotropical didelphids (Araújo et al., 2010; Cantor et al., 2013) suggest that nestedness in their individual‐resource interaction networks followed the “shared preference model” (Svanbäck & Bolnick, 2005), which states that individuals present identical rank of preferable food items, and new resources are added in a predictable order by specialists and generalists individuals. For the marsupial *Didelphis albiventris* in a Neotropical forest, network nestedness emerges during warm‐wet season (when the network is more nested than expected by chance) but not during the cool‐dry season. These results suggest that nestedness structure is broken in low‐resource periods (cool‐dry season), when the similarity of resource use increases between individuals with broader and narrower diets (Cantor et al., 2013). Our study, however, did not show a similar pattern, with no relationship of nestedness with seasonal food resource availability, but rather with habitat structure.

The relationship that we found between habitat structure and nestedness could be ultimately related to the way in which individuals of *G. agilis* use the vertical space in different sites. Spaced and thick trees are habitat features normally present in more structured forests (e.g., mature and pristine forests; Felfili, 1995; Cooper‐Ellis, Foster, Carlton, & Lezberg, 1999; Hitimana, Kiyiapi, & Njunge, 2004), possibly generating a greater vertical space availability (Hitimana et al., 2004) and increasing vertical segregation among individuals. Indeed, we found a general pattern of less use of the understory by *G. agilis* in the site with thin and closer trees (EJBB1; Supporting Information Figures S1, S6 and Table S3). Nestedness in more structured habitats could be enhanced by individuals that explore the ground and the upper strata, potentially adding new food item types (e.g., Erwin, 1995; Aléssio, Pontes, & Silva, 2005; Martins & Gribel, 2007) that are not accessed by individuals that explore exclusively or more frequently the ground level. In other words, more terrestrial individuals would have a diet composed by a subset (narrow dietary niche) of the food items consumed by individuals that use both the ground and the above ground strata (wide dietary niche).

Network variation at different spatio‐temporal scales through species‐based network analysis has been reported in other studies (see Trøjelsgaard & Olesen, 2016). However, to our knowledge, there are no studies testing for direct relatedness of habitat structure and nestedness, especially considering within‐population networks. Tests directly relating the role of spatio‐temporal components on network structure are more common in macroecological studies, showing for example, relationship of annual precipitation, temperature seasonality, and latitude with nestedness (Takemoto, Kanamaru, & Feng, 2014; Trøjelsgaard & Olesen, 2013). At smaller scales, it has been shown (but not tested for direct relationship) that variation in biotic and abiotic factors (e.g., heterogeneity, vegetation productivity, temperature, and precipitation) increases nestedness, playing a larger role in comparison to evolutionary constraints (Robinson, Hauzy, Loeuille, & Albrectsen, 2015; Thompson, Adam, Hultgren, & Thacker, 2013).

Modularity in individual‐resource network context means that there is an organization of distinct groups of individuals specialized on distinct sets of resources. Our results regarding network modularity presented less variation among sites in comparison to the results for nestedness. The four sites presented much lower values of modularity than expected by chance during the cool‐dry season, when mean SES was negative and >7 times higher than in the warm‐wet season. Differently than our initial expectation on a relationship between modularity and habitat structure, our results showed that this network metric was associated with food resource availability (diversity arthropods and fruit abundance), which tended to be lower during the cool‐dry season in all sites (Supporting Information Figures S2 and S3), explaining the general pattern of low modularity in this season.

We detected a lack of association between arthropod dry mass and modularity, which suggests that the overall abundance of this type of food resource is not the preponderant factor that shapes the food–consumer interactions in our study area. These interactions seem to be more influenced by the number and evenness of different items available, as revealed by the direct relationship between modularity and arthropod diversity index. This pattern indicates that an increase in the diversity of food resources leads to an increase in diet segregation generating modules of individual‐resource interactions. A similar pattern was also found in plant‐herbivorous species‐based networks showing greater modularity and herbivorous specialization during periods of high flower richness, potentially lowering competition (López‐Carretero, Díaz‐Castelazo, Boege, & Rico‐Gray, 2014). The association between food resources with modularity found in the present study for *G*. *agilis* could suggest a similar mechanism for avoiding intraspecific competition.

In addition to the greater availability of resources, *G. agilis* populations tend to reach higher densities during the warm‐wet season due to their seasonal pattern of reproduction (end of the cool‐dry season to the middle/end of warm‐wet season; Martins, Bonato, Da‐Silva, et al., 2006). Indeed, the studied populations increase from 87% to 120% (unpubl. data) during the warm‐wet season. Therefore, differently from the “shared preference model” suggested for Neotropical didelphids (Araújo et al., 2010; Cantor et al., 2013), we propose that our results regarding modularity follow the “competitive refuge model”: that is, individuals share the top‐ranked resources differing in the choice of the alternative ones, and specialization arises when resources start to become less available due to increasing forager densities (Svanbäck & Bolnick, 2005). This pattern of feeding specialization during the warm‐wet season is also reinforced by the results for connectance, which was lower in this season and negatively correlated with modularity, and also by the degree distribution results, which indicated that individuals tend to interact with less food resources in the warm‐wet season.

Contrary to the “shared preference model,” which would produce more nested networks, the “competitive refuge model” probably leads to a weak nestedness since the resources are not added by the consumers in a predictable order. This would explain the lack of consistency of nestedness (which was related to habitat structure) and the more consistent pattern regarding modularity (which was related to food resource) for the different population of *G. agilis*. Our findings are in accordance with the study of Lemos‐Costa et al. (2016), which showed that the “competitive refuge model,” and not the “shared preference model,” is the best supported model explaining the network structure of five animal populations.

## 5 CONCLUSIONS

Evaluation of individual‐based networks of the didelphid opossum *G. agilis* showed that network nestedness is related to habitat structure (mainly tree diameter and distance between trees), whereas modularity is related to food resources availability (arthropod diversity and fruit abundance). Under an individual‐based network context, the relationship between nestedness and habitat structure suggests a differential use of the vertical space and resources among individuals, enhancing nestedness. On the other hand, high modularity during the period of high resource availability (warm‐wet season) indicates discrete groups composed by individuals more specialized on distinct sets of resources in comparison to the period of low‐resource availability. Our study also suggests that, differently from the proposed “shared preference model” for didelphid marsupials, *G. agilis* follows the “competitive refuge model.” The present study reinforces the relevance of studies using network approaches to understand individual variation in resource use within populations, and the potentially role of environment components variation to individual‐based network changes.

## CONFLICT OF INTEREST

None declared.

## AUTHOR CONTRIBUTIONS

NFC, JFR, HFMO, and EMV conceived the ideas and designed methodology; NFC and JFR collected the data; NFC and AJAC identified the food items consumed by *G. agilis*; NFC and HFMO analyzed the data; NFC, HFMO, and EMV led the writing of the manuscript. All authors contributed critically to the drafts and gave final approval for publication.

## Data Availability

Datasets on food items consumed by *G. agilis*, food resource availability, and habitat structure are archived and available in the Dryad Digital Repository, http://dx.doi.org/10.5061/dryad.c73p54m.
