# Supplementary material for: Availability of food resources and habitat structure shape the individual‐resource network of a Neotropical marsupial
Source: Ecol Evol. 2019 Mar 21;9(7):3946–57. doi: 10.1002/ece3.5024 (PMC6468053; doi:10.1002/ece3.5024)
Supplement: Supplementary file 1 [file ECE3-9-3946-s001.pdf]

Camargo et al. Availability of food resources and habitat structure shape the individual-resource network of a Neotropical marsupial: Supplementary Information

Table S1. Results of three network metrics in two seasons based on fecal samples of *Gracilinanus agilis* populations in four sites of savanna woodland forest (cerradão) in the Brazilian savanna (Cerrado).

	Cool-dry				Warm-wet			
	FAL	EEJBB1	EEJBB2	EEJBB3	FAL	EEJBB1	EEJBB2	EEJBB3
Nestedness	22.065	13.134	17.454	21.395	16.176	17.436	20.211	19.374
Modularity	0.147	0.100	0.127	0.171	0.305	0.287	0.324	0.311
Connectance	0.282	0.472	0.429	0.349	0.164	0.210	0.194	0.222

Table S2. Results of the Principal Component Analysis (PCA) obtained from eight habitat variables to compare the habitat structure in four savanna woodland sites (cerradão) during the cool-dry and warm-wet season in a Neotropical savanna (Cerrado). Values are the coefficient of the corresponding eigenvectors.

	PC 1	PC 2
Canopy openness	-0.68	-0.38
Diameter of the nearest tree	0.57	-0.57
Distance to the nearest tree	-0.02	-0.75
Height of the nearest tree	0.63	-0.32
Understory obstruction	-0.06	0.19
Herbaceous obstruction	-0.46	0.11
Canopy connectivity	0.71	0.40
Litter depth	0.45	0.14
Eigenvalue	1.94	1.32
Variance explained (%)	30.9	21.0

Table S3. Results of chi-squared tests comparing aboveground captures of *Gracilinanus agilis* among four sites of savanna woodland in the Brazilian Cerrado. Tests were performed using only the first capture of each individual to enhance data independence. Values in the lower diagonal indicate P-values of pairwise test comparisons. Upper diagonal values indicate percentage of captures above ground for each pair of sites compared (line values first). Numbers between parentheses following site name indicate the total number of individuals captured. *p*-values in bold indicate statistical significance ($p \leq 0.05$).

	FAL	EEJBB1	EEJBB2	EEJBB3
FAL (112)	-	58%/43%	58%/56%	58%/63%
EEJBB1 (123)	**0.015**	-	43%/56%	43%/63%
EEJBB2 (109)	0.656	**0.050**	-	56%/63%
EEJBB3 (55)	0.559	**0.011**	0.346	-

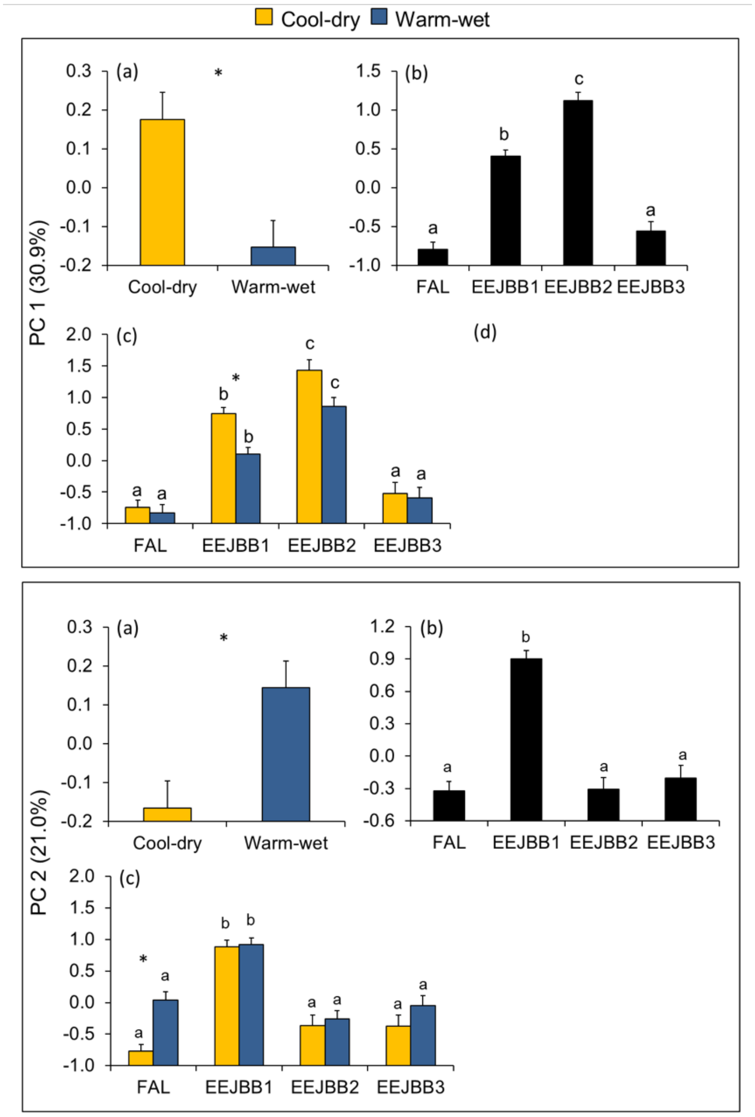

Figure S1. Results of a factorial Multivariate Analysis of Variance (MANOVA) of the two axis scores (PC 1 and PC 2; mean ± SE) obtained in the Principal Component analysis comparing habitat structure between seasons (a; $F_{2,527} = 11.32$, $p < 0.0001$), among sites (b; $F_{6,1054} = 61.26$, $p < 0.0001$) and the interaction between sites and seasons (c; $F_{6,1054} = 3.29$, $p = 0.003$). Asterisks show statistical significance ($p \leq 0.05$) between seasons disregarding sites (a), or considering seasons within each site (c). Different letters above bars indicate differences among sites disregarding seasons (b), or among sites within the same season (c). For more details of the PCA analysis, see Supporting information Figure S6 and Table S2.

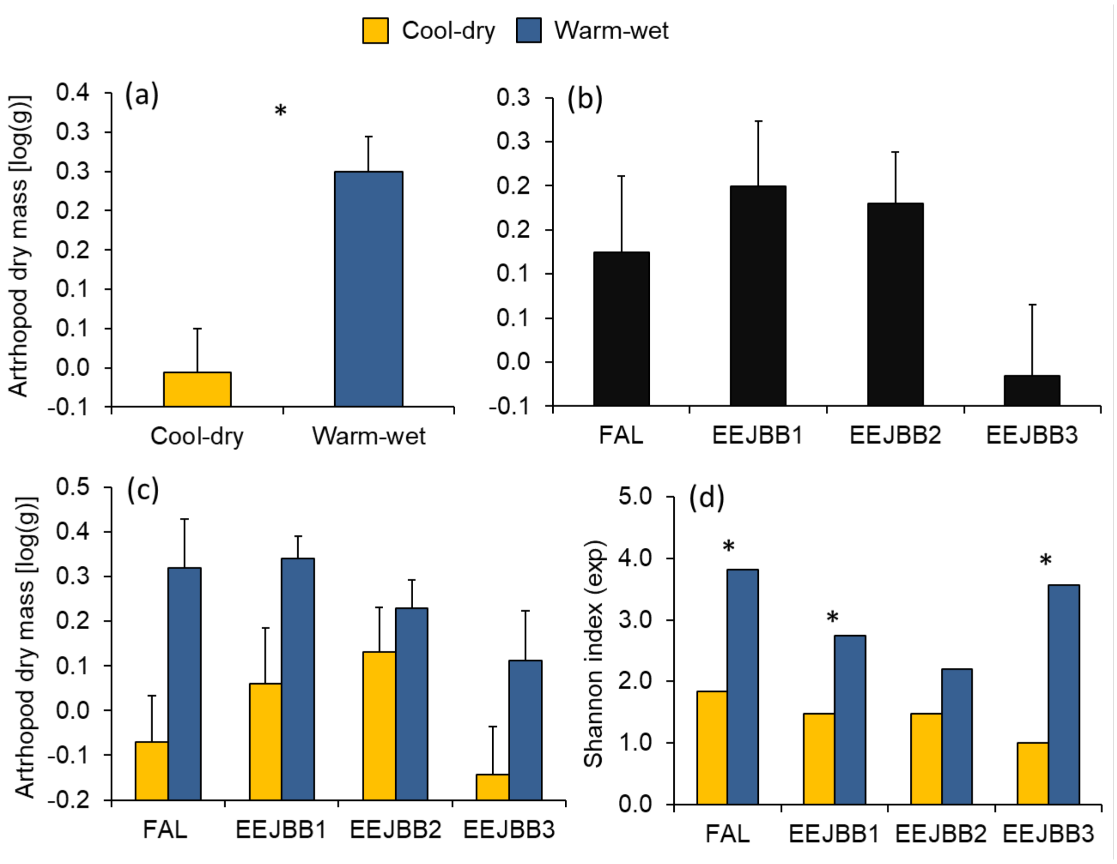

Figure S2. Results of a factorial Analysis of Variance (ANOVA) of arthropod dry mass (mean ± SE) comparing seasons (a), sites (b) and the interaction between sites and seasons (c). The analysis showed differences between seasons ($F_{1,64}$ = 13.25, p = 0.001) but no differences considering sites ($F_{3,64}$ = 1.92, p = 0.135) or the interaction between sites and seasons ($F_{3,64}$ = 0.72, p = 0.542). Figure (d) shows comparisons of arthropod diversity between seasons within each site using a modified t-test for Shannon index. Asterisks show statistical significance ($p \leq 0.05$).

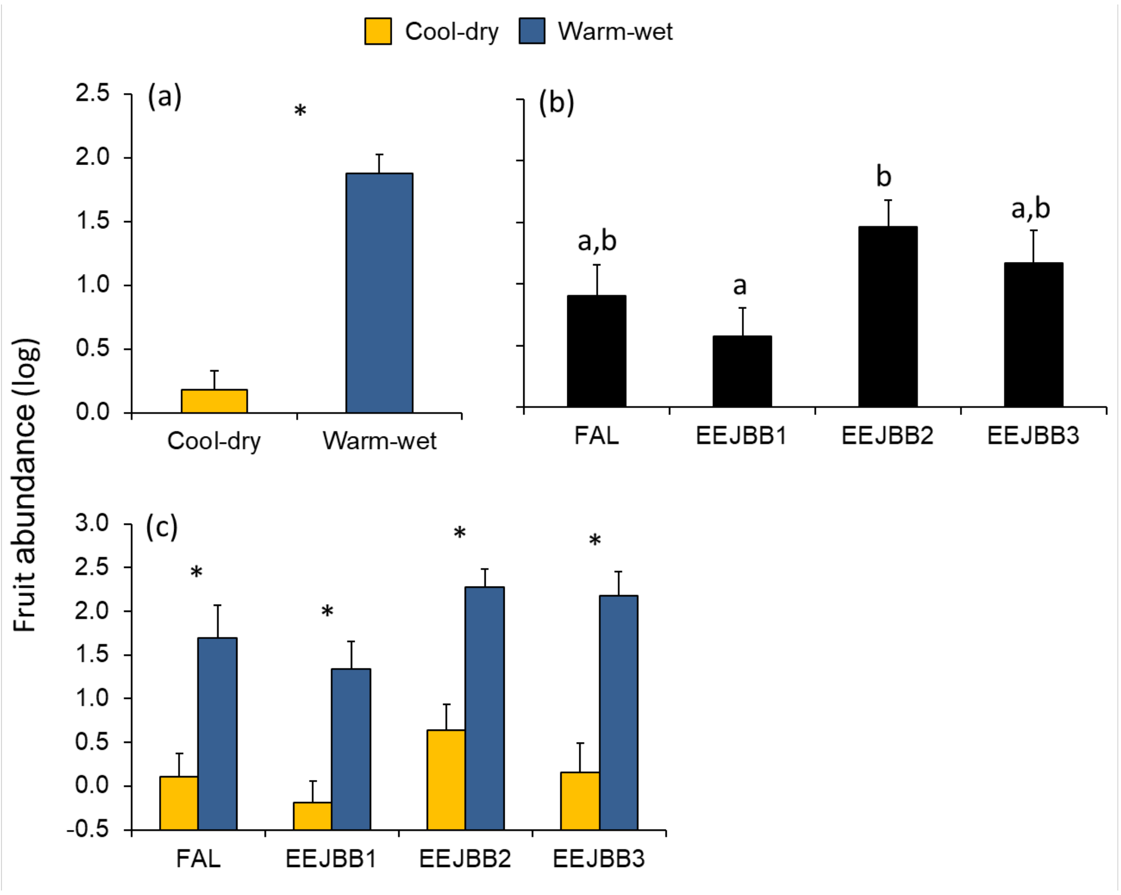

Figure S3. Results of a factorial Analysis of Variance (ANOVA) of fruit abundance (mean ± SE) comparing seasons (a), sites (b) and the interaction between sites and seasons (c). The analysis showed differences between seasons ($F_{1,184}$ = 65.03, p < 0.0001) and among sites ($F_{3,184}$ = 3.23, p = 0.024), but no interaction between sites and seasons ($F_{3,184}$ = 0.29, p = 0.836). Asterisks show statistical significance ($p \leq 0.05$) between seasons and different letters indicate differences between sites.

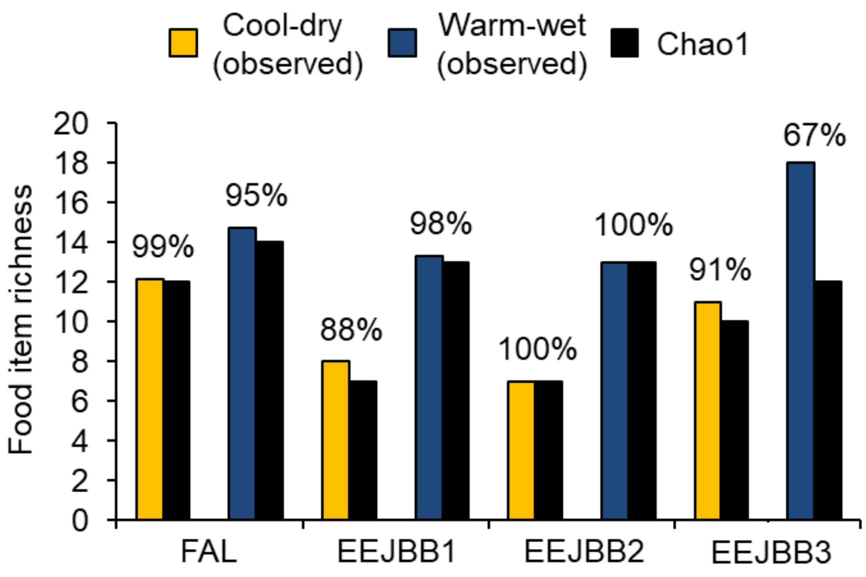

Figure S4. Observed and potential richness (Chao1 estimator of richness) of food items based on fecal samples of *Gracilinanus agilis* populations in four sites of savanna woodland forest (cerradão) in the Brazilian savanna (Cerrado). Values above bars represent the percentage of food items observed according to the potential richness.

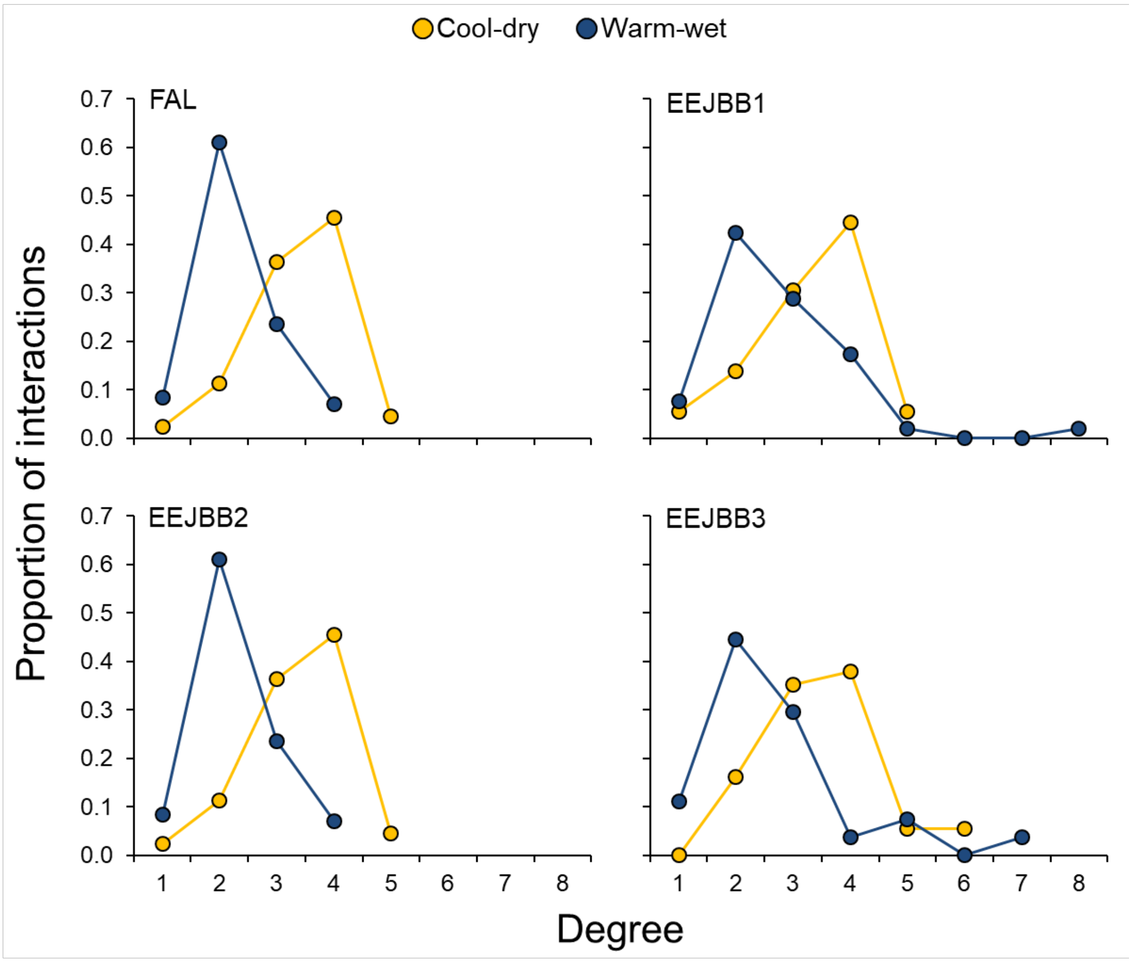

Figure S5. Proportion of individuals that interacted with *n* food items (degree) in the cool-dry and warm wet-seasons based on fecal samples of *Gracilinanus agilis* populations in four sites of savanna woodland forest (cerradão) in the Brazilian savanna (Cerrado).

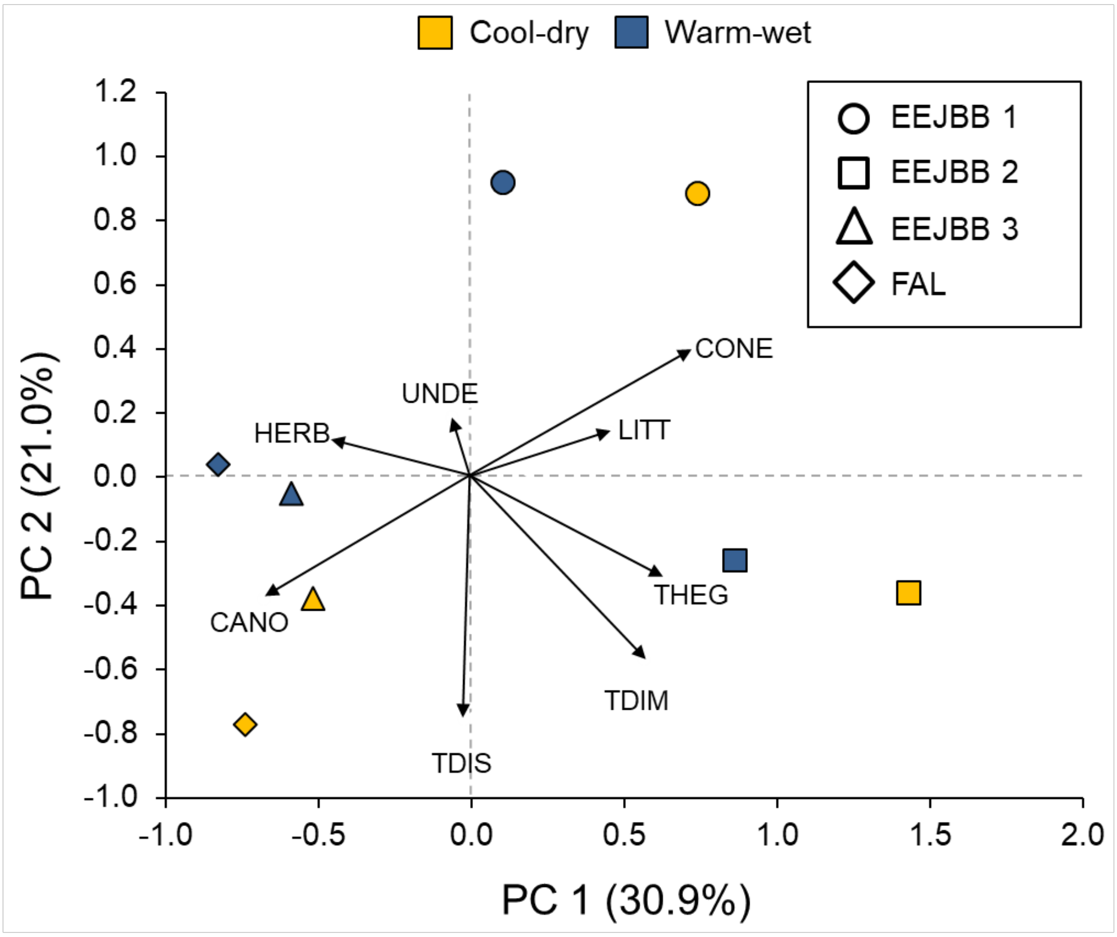

Figure S6. Results of the principal component analysis (PCA) of habitat structure variables obtained during the cool-dry and warm-wet seasons in four savanna woodland (cerradão) sites in the Brazilian savanna (Cerrado). CANO = canopy openness; CONE = canopy connectivity; HERB = herbaceous obstruction; LITT = litter depth; TDIM = diameter of the nearest tree; TDIS = distance to the nearest tree; THEG = height of the nearest tree; UNDE = understory obstruction. The percentage of the total variance explained by each principal component is indicated between parentheses. Eigenvector coefficients can be found in the Table S2.
